# Contrasts among cationic phytochemical landscapes in the southern United States

**DOI:** 10.1002/pei3.10093

**Published:** 2022-10-04

**Authors:** Luis Y. Santiago‐Rosario, Kyle E. Harms, Dylan Craven

**Affiliations:** ^1^ Department of Biological Sciences Louisiana State University Baton Rouge Louisiana USA; ^2^ Centro de Modelación y Monitoreo de Ecosistemas Facultad de Ciencias, Universidad Mayor Santiago de Chile Chile

**Keywords:** calcium, homeostasis, magnesium, potassium, Random Forest, sodium, spatial autocorrelation

## Abstract

Understanding the phytochemical landscapes of essential and nonessential chemical elements to plants provides an opportunity to better link biogeochemical cycles to trophic ecology. We investigated the formation and regulation of the cationic phytochemical landscapes of four key elements for biota: Ca, Mg, K, and Na. We collected aboveground tissues of plants in *Atriplex*, *Helianthus*, and *Opuntia* and adjacent soils from 51, 131, and 83 sites, respectively, across the southern United States. We determined the spatial variability of these cations in plants and soils. Also, we quantified the homeostasis coefficient for each cation and genus combination, by using mixed‐effect models, with spatially correlated random effects. Additionally, using random forest models, we modeled the influence of bioclimatic, soil, and spatial variables on plant cationic concentrations. Sodium variability and spatial autocorrelation were considerably greater than for Ca, Mg, or K. Calcium, Mg, and K exhibited strongly homeostatic patterns, in striking contrast to non‐homeostatic Na. Even so, climatic and soil variables explained a large proportion of plants' cationic concentrations. Essential elements (Ca, Mg, and K) appeared to be homeostatically regulated, which contrasted sharply with Na, a nonessential element for most plants. In addition, we provide evidence for the No‐Escape‐from‐Sodium hypothesis in real‐world ecosystems, indicating that plant Na concentrations tend to increase as substrate Na levels increase.

## Graphical abstract

Plants are key conduits that link substrate to higher trophic levels in food webs and biogeochemistry cycles. In this study, we set out to characterize how the phytochemical landscape of cations is formed and maintained across the southern United States and show some insights into how essential (Ca, Mg and K) and nonessential (Na) elements for plants, are distributed across in tissues across a heterogeneous landscape. We also show how plants homeostatically regulate essential elements regardless of high substrate variation, a pattern contrary to that of nonessential elements like Na. We also show field evidence for the No‐Escape‐from‐Sodium hypothesis indicating that plant Na concentrations tend to increase as substrate Na levels increase.

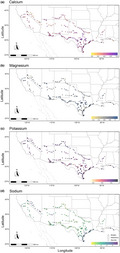

## 1 INTRODUCTION

As essential components of communities and contributors to ecosystem functions, plants and other primary producers represent key conduits that link substrates to higher trophic levels in food webs and biogeochemical cycles (Austin & Zanne, 2015; Farago, 1994; Kaspari & Powers, 2016; Sterner & Elser, 2002; Wang et al., 2007; Waring et al., 2015; Welti et al., 2017). Plants make soil‐borne elements available to herbivores and other consumers, often while altering their relative proportions, that is, the ecological stoichiometry of those elements (Hunter, 2016; Sterner & Elser, 2002). Thus, the formation and maintenance of the phytochemical landscape, as envisioned by Hunter (2016), strongly influences plant‐herbivore interactions, community assembly, and ecosystem dynamics across landscapes that differ in climatic conditions, soil composition, and other properties (e.g, Filipiak & Weiner, 2017; Mitchell et al., 2020; Moore et al., 2010; Stallard & Edmond, 1981; Zhang et al., 2012). Yet, our understanding of the formation, composition, and function of phytochemical landscapes of many biotically important elements remains relatively understudied, especially for elements like sodium (Na) and other influential cations (Hunter, 2016; Kaspari, 2021; Kaspari & Powers, 2016).

As an often biologically critical element, Na is distinctive. It is generally considered nonessential for most plants, yet it is a key and essential nutrient for animals and decomposers (Clay et al., 2014; Kaspari, 2020; Kaspari, 2021; Kronzucker et al., 2013). The distribution of Na across terrestrial habitats is exceptionally heterogeneous. Dry or xeric habitats, certain geological formations (e.g., salt deposits, salt domes, sodium feldspar rocks), and coastal habitats often have the highest concentrations of environmentally available Na ions (Kapustina, 2001; Kaspari, 2020; Martin et al., 2010; National Atmospheric Deposition Program [NRSP‐3], 2020; Smith, 2013; Stallard & Edmond, 1981). High variation and shortfalls in environmental Na have important consequences for organismal behavior, physiological performance, species interactions, and community assembly (e.g., Borer et al., 2019; Bradshaw & Bradshaw, 1999; Bravo et al., 2012; Brewer & Grace, 1990; Clay et al., 2014; Clay et al., 2022; Prather et al., 2018; Snell‐Rood et al., 2014). Therefore, determining the extent to which plant Na concentrations are coupled with those of soils is essential for deepening our understanding of how community‐ and ecosystem‐level processes vary across space.

Sodium is considered nonessential for the development of most plants (Grigore et al., 2012; Kronzucker et al., 2013). Notable exceptions in which Na benefits development or performance include most halophytes (Cheeseman, 2015; Flowers & Colmer, 2008; Kanai & Sakai, 2021) in certain environmental conditions, including specific ranges of Na concentration in the substrate (Santiago‐Rosario et al., 2021). Certain C_4_ (photosynthesis via C_4_ carbon fixation or the Hatch‐Slack pathway) and crassulacean acid metabolism (CAM) plants benefit ‐ at specific substrate concentrations ‐ from slight increases in substrate Na (Furumoto et al., 2011; Subbarao et al., 2003). Some C_4_ plant species in the families Amaranthaceae, Asteraceae, Brassicaceae, Cyperaceae, Fabaceae, Poaceae, Portulacaceae, and Solanaceae, among others, found at relatively low concentrations of substrate Na, benefit from slight increases in Na by increasing biomass yield and reducing chlorosis (Brownell & Crossland, 1972; Johnston et al., 1988; Pessarakli & Marcum, 2000). For example, for species in the genus *Flaveria* (Asteraceae), Na is an essential nutrient as a transporter required for C_4_ photosynthesis (Furumoto et al., 2011). Additionally, Na increased growth in the CAM species *Bryophyllum delagoense* (Crassulaceae) when substrate Na was increased to 0.1 meq/L NaCl as compared to individuals in basal culture solution (0.07 μeq/L NaCl), especially when grown under conditions of short‐day length and high diurnal temperature variation (Brownell & Crossland, 1974). Therefore, at certain low concentrations of substrate Na, slight increases in Na appear to positively influence some C_4_ and CAM plants' growth (Subbarao et al., 1999; Subbarao et al., 2003). However, it is important to note that whether Na′s effect on these species results from drought adaptations or metabolic micronutrient functions remains unresolved among plant physiologists (Brownell, 1968; Subbarao et al., 2003).

The No‐Escape‐from‐Sodium hypothesis posits that plants' tissues broadly increase in Na concentration as the concentration of Na in the substrate or solution increases, irrespective of their growth responses, and there is empirical support for this pattern across selected plant taxa (Santiago‐Rosario et al., 2021). However, our understanding of how plants respond to increasing substrate Na comes mostly from controlled laboratory and greenhouse experiments, which may or may not align with patterns in real‐world ecosystems. In the current study, we tested the No‐Escape‐from‐Sodium hypothesis in the field across the southern continental United States. We also included three additional cations: calcium (Ca), magnesium (Mg), and potassium (K), because of the essential role they play in plant physiology and ecosystem processes and their ubiquitous distribution across the soilscape. We aimed to characterize the phytocationic landscapes for these four cations, by identifying the potential environmental drivers of plant cation concentrations and asking whether there is evidence for homeostatic regulation.

## 2 METHODS

### 2.1 Plant taxa, field collections, and elemental analysis

We selected the plant genera *Atriplex* (saltbushes), *Helianthus* (sunflowers), and *Opuntia* (prickly pears), based on their contrasting photosynthetic pathways and their widespread distributions across the conterminous United States. *Atriplex* comprises 65 North American species that use the C_4_ photosynthetic pathway (Brown, 1956; Kadereit et al., 2010). *Helianthus* species use the C_3_ photosynthetic pathway and include over 49 species across North America (Heiser et al., 1969; Timme et al., 2007; Vanaja et al., 2011). Finally, *Opuntia* comprises ~110 species in North America, with all species using the CAM photosynthetic pathway (Cui & Nobel, 1994; Majure et al., 2012).

**FIGURE 1:**
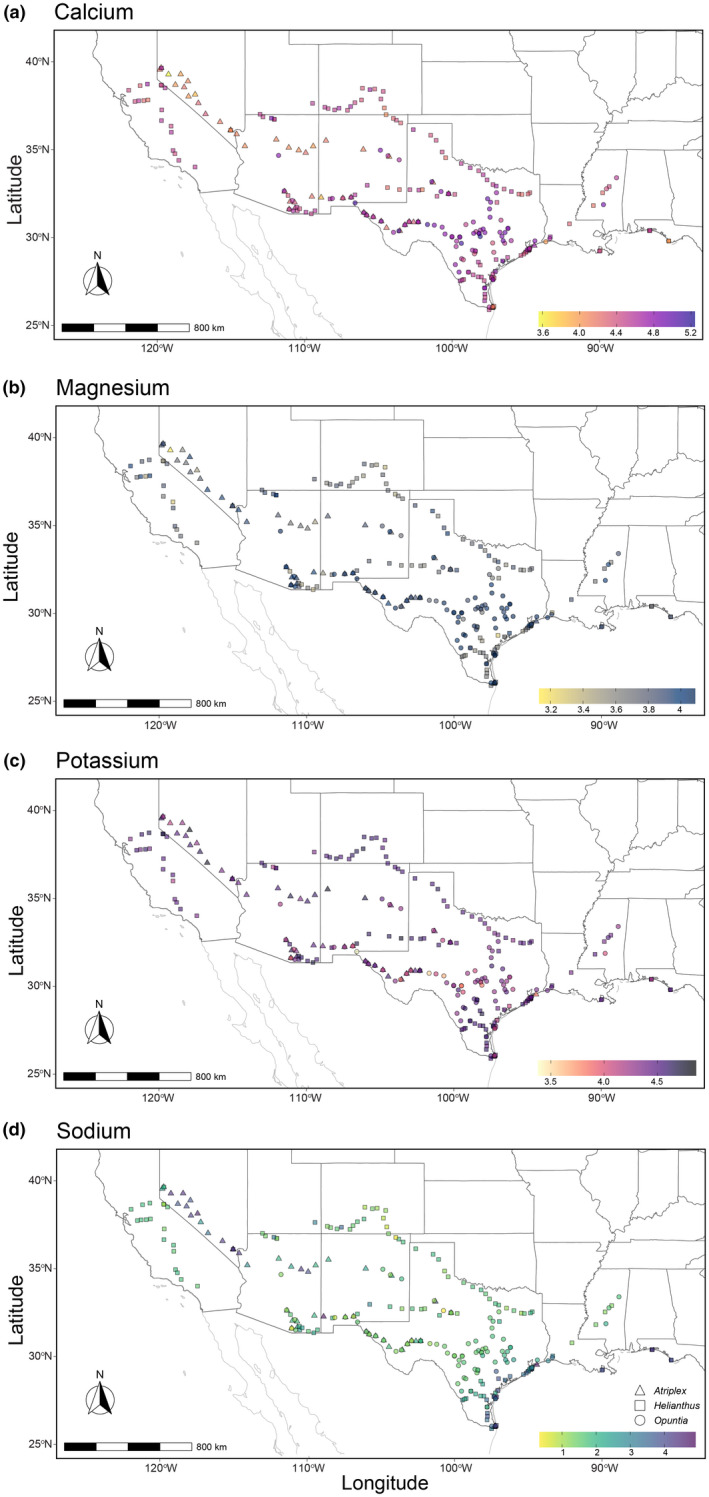
Geographic locations and aboveground phytochemical landscapes of (a) Ca, (b) K, (c) Mg, and (d) Na (log_10_ ppm) across the southern United States. Triangle, square, and circle shapes depict sites where *Atriplex*, *Helianthus*, and *Opuntia* were sampled, respectively. A color gradient demonstrates plant Ca, K, Mg, and Na concentrations, with darker shades indicating higher concentrations and lighter shades indicating lower concentrations.

We collected *Atriplex*, *Helianthus*, and *Opuntia* aboveground photosynthetic tissues in 51, 131, and 83 sites, respectively. Collection sites across the southern U.S. ranged from Florida (~86° W) to California (~123° W). Sampling was completed during the summers of 2018, 2019, and 2020 (Figure 1). We collected samples of aboveground tissues (leaves and stems), adjacent soil (top 10 cm), and one voucher specimen (for genus verification) from each site, along with GPS coordinates.

Plant and soil samples were oven‐dried at 65°C for 7 days. Aboveground tissues (i.e., leaves for *Atriplex* and *Helianthus*, and cladodes for *Opuntia*) were processed and ground into a fine, homogeneous powder for each site. Dried soil samples were passed through a 2 mm copper sieve to remove rocks and organic debris. Concentrations of Ca, Mg, K, Na, and P on a dry mass basis for soils and plant tissues were determined at the Soil Testing and Plant Analysis Laboratory at Louisiana State University (http://www.lsuagcenter.com), using inductively coupled plasma with atomic emission spectrometry (ICP‐AES) following standard protocols (Munns et al., 2010). Soil pH (1:1 water) was also measured.

### 2.2 Abiotic conditions

Bioclimatic and elevation data were extracted for each sample site using the ‘raster’ package (Hijmans & van Etten, 2022) with a resolution of 4.6 km^2^ in R (R Core Team, 2020). Climatic variables were mean annual temperature (MAT, °C), mean diurnal temperature range, temperature seasonality, annual precipitation (mm), precipitation in the wettest month (mm), precipitation in the driest month (mm), and precipitation seasonality (coefficient of variation). We also measured the distance from each site to its nearest relevant coast (km), as a proxy for its proximity to its effective marine source of cations (as in Bravo & Harms, 2017). We used the Gulf of Mexico‐Pacific Continental Divide, as wind movement and precipitation for both sides of the divide are associated more closely with their respective oceanic sources (Adams & Comrie, 1997). For each sample location, we expanded a circle using the Google Earth (http://www.google.com/earth) circumference tool until the edge of the circle first contacted the relevant coast. The radius of the circle was recorded as the effective distance to the nearest marine source for cations.

### 2.3 Data analysis

#### 2.3.1 Cation variability and spatial autocorrelation analysis

We performed a paired t‐test to compare cation concentrations in aboveground plant tissues and adjacent soils and calculated the coefficient of variation to represent variation across space. To quantify whether inter‐site proximity influenced cation concentration similarities for each element for aboveground plant tissues in each genus, as well as adjacent soils, we performed a Mantel test using the package ‘ecodist’ (Goslee & Urban, 2007). All cation concentrations were log_10_ transformed and compared from site to site in a pairwise manner. All values were compiled into a matrix of differences (i.e., values closer to zero indicate more significant similarity in cation composition), and the absolute value was calculated to remove all negative values from the matrix. Additionally, we calculated the Haversine distance (km) among sites using the package ‘*geosphere*’ (Hijmans et al., 2019).

We performed Mantel tests to assess whether: (1) plants nearby shared similar cation concentrations, and (2) soils nearby shared similar cation concentrations. For a Mantel test, a significant result reveals that distances between two matrices are correlated (Rossi, 1996). Correlations can be positive or negative, representing how the variables are associated. Correlation in the ‘ecodist’ package is calculated using a Spearman approach, and all Mantel test calculations were performed using 9999 permutations. We also conducted a Mantel correlogram using the R function ‘*mgram*’ of the package ‘ecodist’ by dividing the data into 20 distance classes. For each of the three types of tests (1 and 2 above) a Mantel correlogram was performed for each cation and genus combination.

### 2.4 Abiotic drivers of plant cation concentrations

To quantify the relative potential importance of abiotic conditions in determining plant cation concentrations, we fitted spatial regression models for each combination of plant genus and cation (12 models = 3 plant genera *x* 4 plant cations) with the Random Forest algorithm using the packages ‘spatialRF’ and ‘ranger’ (Benito, 2021; Wright & Ziegler, 2017). For each combination, we considered the following explanatory variables: MAT, temperature seasonality, mean annual precipitation, precipitation in the wettest months, precipitation in the driest months, precipitation seasonality, effective distance to coast, elevation, soil pH, and soil cation (i.e., the same as the plant cation). As multi‐collinearity may affect the interpretation of variable importance of random forest models (Strobl et al., 2007), we estimated variance inflation (VIF) and correlation among abiotic variables and subsequently selected those whose VIF was lower than 4 and whose correlation with other abiotic variables was less than 0.7 (Dormann et al., 2013). We then tested the residuals of a non‐spatial random forest model for spatial autocorrelation using multiscale Moran's I, and, if spatial autocorrelation was statistically significant, fitted spatial Random Forest models with Moran's eigenvector maps to build spatial predictors using the R function ‘*rf_spatial*’. Because the default hyperparameters may not be adequate for each dataset, we used the R function ‘*rf_tuning*’ to select the optimal values for the number of regression trees in the forest, the number of variables to choose from on each tree split, and a minimum number of cases on a terminal node for each model. We repeated each model 50 times using the optimal hyperparameter values with the R function ‘*rf_repeat’*, because Random Forest is a stochastic algorithm whose variability may influence the interpretation of variable importance scores and response curves. We used median R‐squared, calculated as the squared correlation between observed and predicted values, and normalized root mean square errors (NRMSE) to assess model fit across the 50 iterations of each model. We log_10_ transformed plant and soil Na concentrations to normalize their distributions and only fitted a spatial random forest model for *Opuntia* and Na, since that was the only genus and cation combination for which it was required for our Random Forest analyses.

### 2.5 Assessment of homeostasis

To assess the potential for plants to regulate cations in the field, we calculated the homeostasis coefficient *Η* (eta) for each cation and genus combination, as outlined in Sterner and Elser (2002). For this approach, we calculated a stoichiometric ratio based on phosphorus (*P*) concentration for plants and soils. Then the homeostasis coefficient (*H*) was calculated using the modified formula:

$$\log\;y=\log\;\;c+\left(\frac{\log\;x}{H}\right)$$

where *y* is the plant stoichiometric cationic ratio, *x* is the cationic soil ratio, and *c* is a constant. By plotting the logarithms of the plant versus soil stoichiometry, Sterner and Elser (2002) advise that slopes (1/*H*) between 1 and 0 indicate a continuum in homeostatic regulation with a slope value of 0 indicating strict homeostasis and a value of 1 indicating a lack of homeostasis between plant stoichiometry relative to soil stoichiometry (Meunier et al., 2014). However, as cations may be spatially autocorrelated, we accounted for any spatial autocorrelation using the package ‘spaMM’ (Rouseet & Ferdy, 2014). As a statistical approach, ‘spaMM’ fits mixed‐effect models and permits the inclusion of spatial autocorrelation (i.e., Matern) as a random effect. The models generated in this study considered plant cation (denominated as X) stoichiometry (X_plant_:P_plant_) and soil cation stoichiometry (X_soil_:P_soil_) as fixed effects, and spatial autocorrelation was specified as a Matern random effect. For all models, we included spatial autocorrelation as a random effect, and fitted models using a *nu* of 0.5 to keep constancy across the cations and genera considered. All analyses were performed in R Studio (R Core Team, 2020).

## 3 RESULTS

### 3.1 Phytochemical landscapes differ across cations

**FIGURE 2:**
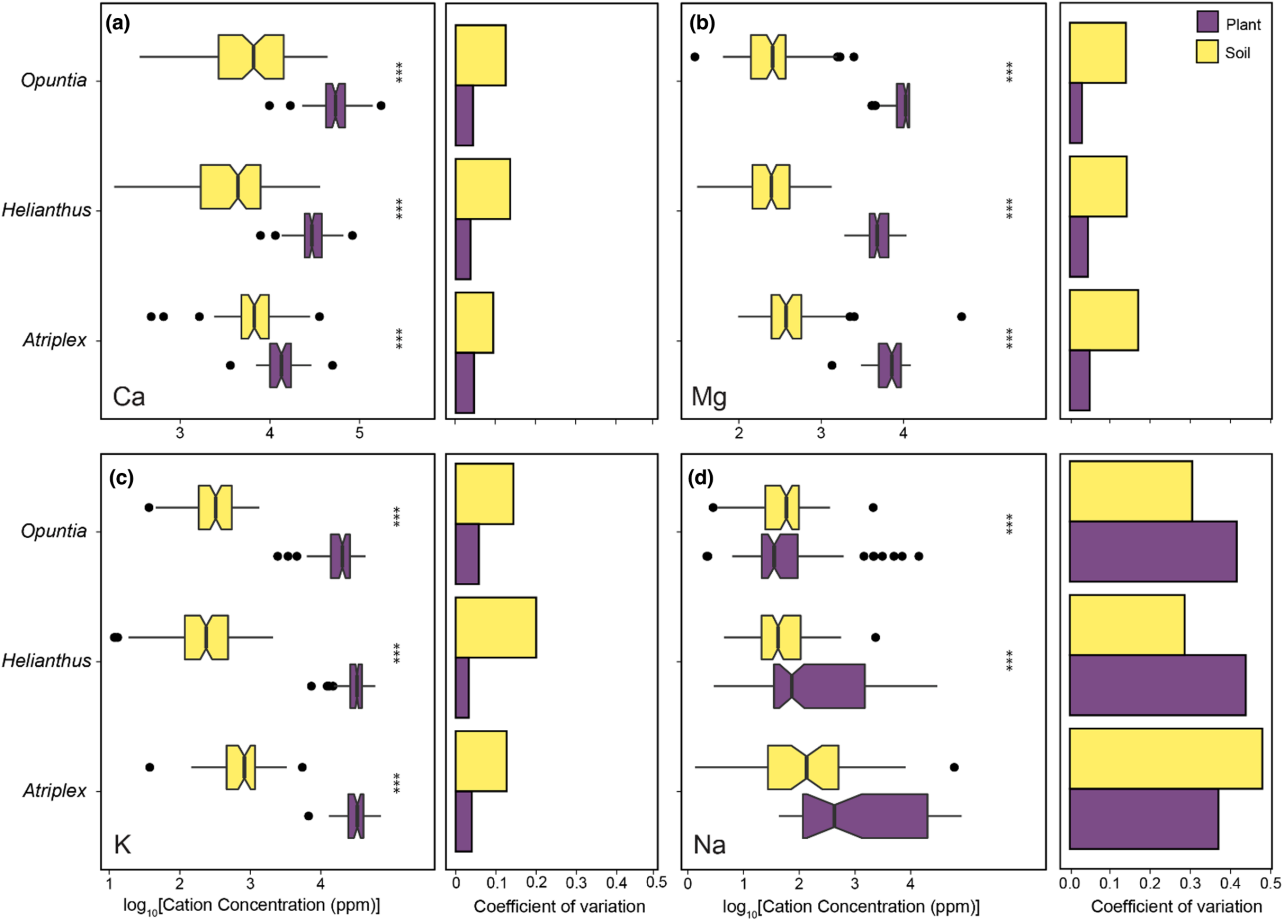
Concentrations and variation in concentrations of cations for aboveground plant tissues (purple) and adjacent soils (gold) for each genus across all sites sampled. Responses for (a) Ca, (b) Mg, (c) K, and (d) Na are shown in boxplots and the coefficients of variation are shown in bar graphs for each genus considered. All responses are log‐transformed. Asterisks (***) indicate a significant difference (*p* < .0001).

Aboveground plant cation concentrations vary to differing degrees across the southern United States and among plant genera sampled. Calcium (Figures 1a and 2a), Mg (Figures 1b and 2b), and K (Figures 1c and 2c) plant tissue concentrations displayed overall low inter‐plant spatial variation. In contrast, Na exhibited considerably higher variation in plant tissue concentrations across all genera sampled (Figures 1d and 2d).

Generally, aboveground plant tissues contained higher cation concentrations than adjacent soils (Figure 2, Table 1). The only exception was Na in *Opuntia*, in which there was no significant difference in aboveground tissue (1.729 log_10_[ppm] ± 0.08) and adjacent soil (1.674 log_10_[ppm] ± 0.06) concentrations (*t*_[82]_ = 0.750, *p* = .453; Figure 2, Table 1). Additionally, Na was the most variable cation across sites in soils and plant tissue samples (Figure 2). For Na in *Atriplex*, soils were more variable than aboveground tissues, contrasting with the patterns observed in *Helianthus* and *Opuntia* (Figure 2d). Strikingly, variation in plant tissues of Ca, Mg, and K was minimal; more variation occurred in the soils found across the sampled landscape for these cations (Figure 2).

**TABLE 1:** Paired t‐test results for plant and soil cation concentrations [log_10_ (ppm)] with a summary of means and standard errors for each genus

		*Atriplex*				*Helianthus*				*Opuntia*			
Cation	Sample	Mean	SE	*t*‐stat	*p*‐value	Mean	SE	*t*‐stat	*p*‐value	Mean	SE	*t*‐stat	*p*‐value
Ca	Plant	4.13	0.03	6.799	**<.0001**	4.47	0.01	20.898	**<.0001**	4.73	0.02	19.866	**<.0001**
	Soil	3.83	0.05			3.60	0.04			3.77	0.05		
Mg	Plant	3.86	0.03	17.782	**<.0001**	3.69	0.01	43.486	**<.0001**	3.97	0.01	44.171	**<.0001**
	Soil	2.64	0.06			2.37	0.03			2.41	0.04		
K	Plant	4.50	0.03	34.736	**<.0001**	4.49	0.01	55.313	**<.0001**	4.26	0.03	36.492	**<.0001**
	Soil	2.87	0.05			2.36	0.04			2.50	0.04		
Na	Plant	3.13	0.16	7.363	**<.0001**	2.27	0.09	6.861	**<.0001**	1.73	0.08	0.750	.455
	Soil	2.07	0.14			1.69	0.04			1.67	0.06		

*Note*: Significant results are in bold.

### 3.2 Spatial autocorrelation is generally stronger for plant Na than the other cations across all genera

**FIGURE 3:**
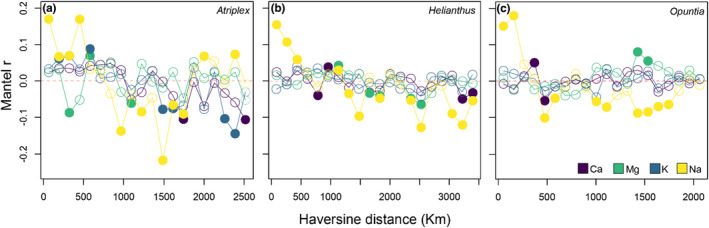
Mantel correlograms for plant cation spatial structure for *Atriplex*, *Helianthus*, and *Opuntia*. Mantel r values are plotted on the y‐axis. Closed circles indicate significant correlations (*p* < .05) after 9999 permutations. Positive correlations indicate positive spatial autocorrelation among sites.

Aboveground plant Ca, Mg, and K exhibited generally low spatial autocorrelation across genera, especially focusing on positive spatial autocorrelation at distances <1000 km (Figure 3). *Atriplex* had weak overall autocorrelation for plant Ca concentrations (*r* = 0.094, *p* = .043), and somewhat stronger autocorrelation for K concentrations (*r* = 0.154, *p* = .004; Table 2). *Helianthus* also had weak overall autocorrelation in K concentrations (*r* = 0.054, *p* = .050; Table 2). In contrast, there was strong significant overall autocorrelation for plant Na concentrations across all three genera (*p* < .0001; Table 2). On average, the correlograms for Na were decreasing, with positive significant spatial autocorrelation found for distances up to ~482 km for *Atriplex*, ~432 km for *Helianthus*, and ~ 162 km for *Opuntia*.

**TABLE 2:** Mantel test results summary for each genus and cation combination

		Aboveground plant cation similarity spatial structure		Soil cation similarity spatial structure	
Cation	Genus	Mantel r	*p*‐value	Mantel r	*p*‐value
Ca	*Atriplex*	**0.094**	**.043**	0.077	.086
	*Helianthus*	0.022	.222	**0.052**	**.026**
	*Opuntia*	−0.023	.654	0.051	.113
Mg	*Atriplex*	−0.013	.569	**0.201**	**.001**
	*Helianthus*	0.044	.052	0.036	.095
	*Opuntia*	−0.028	.674	0.012	.384
K	*Atriplex*	**0.154**	**.004**	0.065	.117
	*Helianthus*	**0.054**	**.050**	**0.056**	**.022**
	*Opuntia*	0.034	.254	0.068	.089
Na	*Atriplex*	**0.268**	**.0001**	**0.119**	**.009**
	*Helianthus*	**0.223**	**.0001**	−0.022	.816
	*Opuntia*	**0.282**	**.0001**	0.041	.209

*Note*: Significant results are in bold.

**FIGURE 4:**
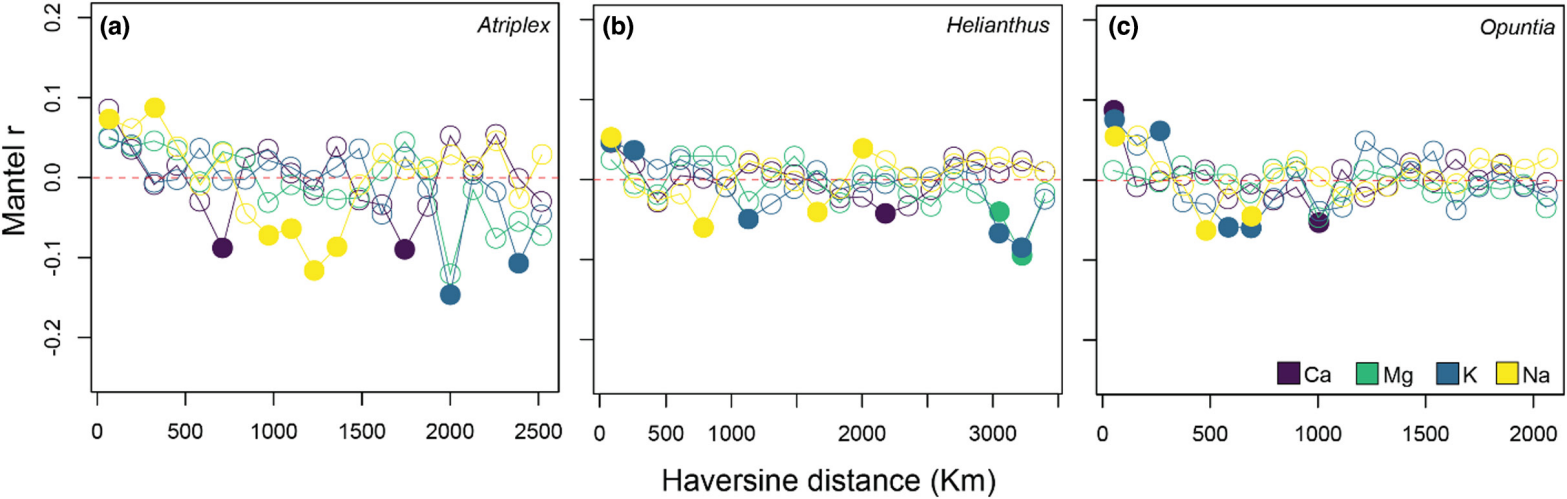
Mantel correlograms for soil cation spatial structure for *Atriplex*, *Helianthus*, and *Opuntia*. Mantel r values are plotted on the y‐axis. Closed circles indicate significant correlations (*p* < .05) after 9999 permutations. Positive correlations indicate positive spatial autocorrelation among sites.

Soils, on average, exhibited varied spatial autocorrelation among the cations. For *Atriplex* sites, Mg (*r* = 0.201, *p* = .001) and Na (*r* = 0.119, *p* = .009) revealed stronger overall autocorrelation (Table 2). For *Helianthus* sites, Ca (*r* = 0.052, *p* = .026) and K (*r* = 0.056, *p* = .022) displayed weak autocorrelation (Table 2). *Opuntia* sites exhibited no significant overall autocorrelation for any cations considered (Figure 4c, Table 2).

### 3.3 Homeostasis coefficients vary across cations and taxa

For Ca, *H* values differed across genera, with *Helianthus* expressing a much more homeostatic pattern (*H* = 31.54) as compared to *Atriplex* (*H* = 4.45); *Opuntia* expressed the lowest homeostatic pattern (*H* = 2.92; Table 3). For Mg, *Helianthus* had the highest *H* value (*H* = 15.35), followed by *Atriplex* (*H* = 8.59) and *Opuntia* (*H* = 4.28; Table 3). The homeostasis coefficient for K was more comparable among genera than for Ca and Mg; *Helianthus* (*H* = 5.54) had the highest *H* value, followed by *Atriplex* (*H* = 5.41) and *Opuntia* (*H* = 5.33; Table 3).

**TABLE 3:** Homeostasis coefficient results (*H*) for all genera and cations sampled. Results are given as slopes and *H* along with their confidence intervals and t‐values

Genus	Cation	Slope	SE_slope_	*t*‐value	95% CI_slope_ lower	95% CI_slope_ upper	*H*	95% CI_*H*_ lower	95% CI_*H*_ upper
*Atriplex*	Ca	0.22	0.05	4.41	0.122	0.326	4.45	8.14	3.06
	Mg	0.12	0.06	1.79	0.013	0.252	8.59	74.52	3.97
	K	0.18	0.08	2.40	0.011	0.350	5.41	91.74	2.86
	Na	0.54	0.09	5.47	0.289	0.755	1.85	3.46	1.32
*Helianthus*	Ca	0.03	0.03	1.09	0.026	0.089	31.54	37.88	11.14
	Mg	0.07	0.04	1.62	0.015	0.145	15.35	67.43	6.90
	K	0.18	0.03	5.39	0.115	0.248	5.54	8.69	4.03
	Na	0.40	0.08	4.68	0.228	0.564	2.53	4.39	1.77
*Opuntia*	Ca	0.34	0.06	5.66	0.222	0.463	2.92	4.49	2.16
	Mg	0.23	0.05	4.27	0.121	0.345	4.28	8.29	2.90
	K	0.19	0.48	3.94	0.084	0.295	5.33	11.90	3.39
	Na	0.38	0.09	4.16	0.191	0.581	2.61	5.25	1.72

The homeostasis coefficient for Na followed a different pattern than Ca, Mg, and K, with consistently low values for all genera. *Opuntia* had the marginally highest *H* value (*H* = 2.61), followed by *Helianthus* (*H* = 2.53) and *Atriplex* (*H* = 1.85; Table 3).

### 3.4 Soil cations and abiotic variables explain most of the variation in plant cation concentrations

**FIGURE 5:**
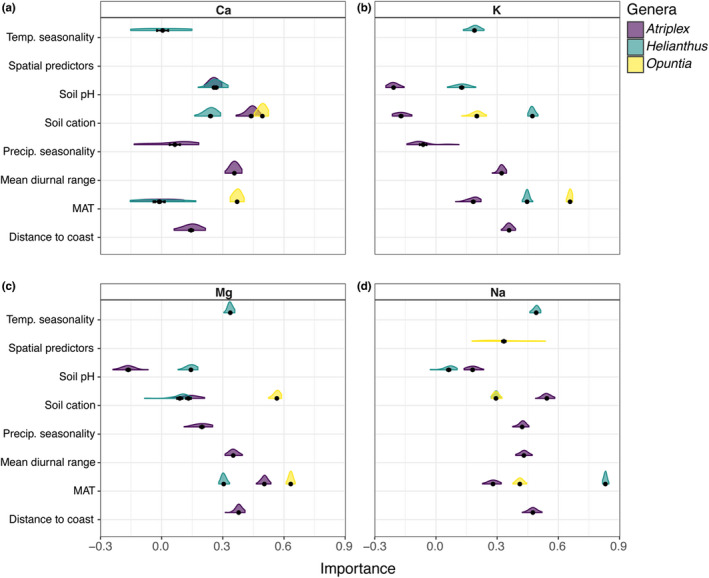
Importance values of potential abiotic drivers of plant concentrations of (a) Ca (b) K (c) Mg and (d) Na for *Atriplex, Helianthus*, and *Opuntia*. Abiotic drivers are ranked by relative variable importance calculated for each random forest model; variable importance represents the increase in mean error across trees when a predictor is permuted. Black points and error bars are mean and 95% confidence values of relative importance values and were calculated across 50 iterations of the same random forest model. For visualization purposes, the relative importance scores of spatial predictors were excluded.

**FIGURE 6:**
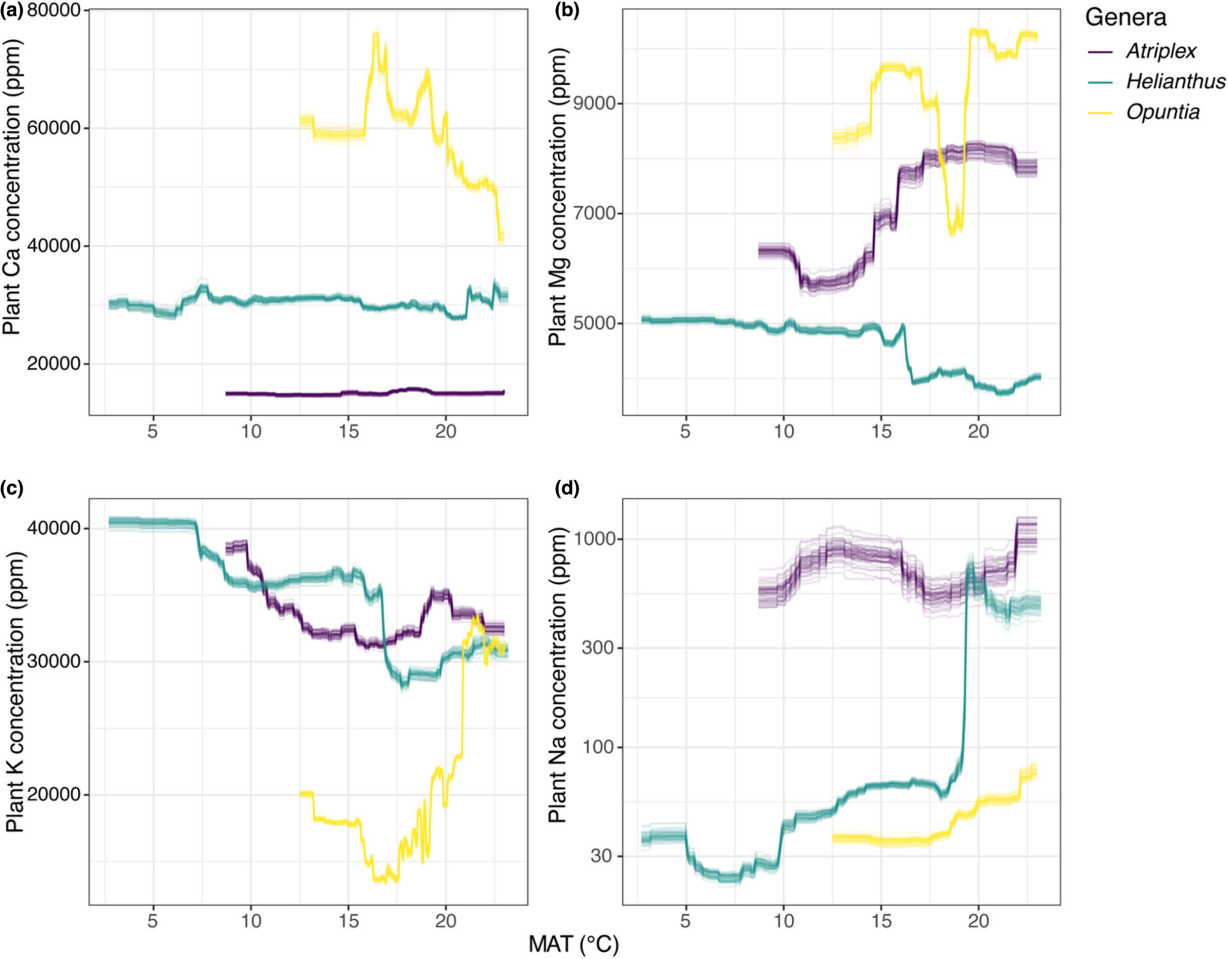
Responses of plant concentrations of (a) Ca, (b) Mg, (c) K and (d) Na for *Atriplex, Helianthus*, and *Opuntia* to mean annual temperature (MAT). Each line represents a response curve estimated by a random forest model, which was fitted for each combination of plant genus and cation and repeated 50 times. Plant Na is on a log_10_ scale.

**FIGURE 7:**
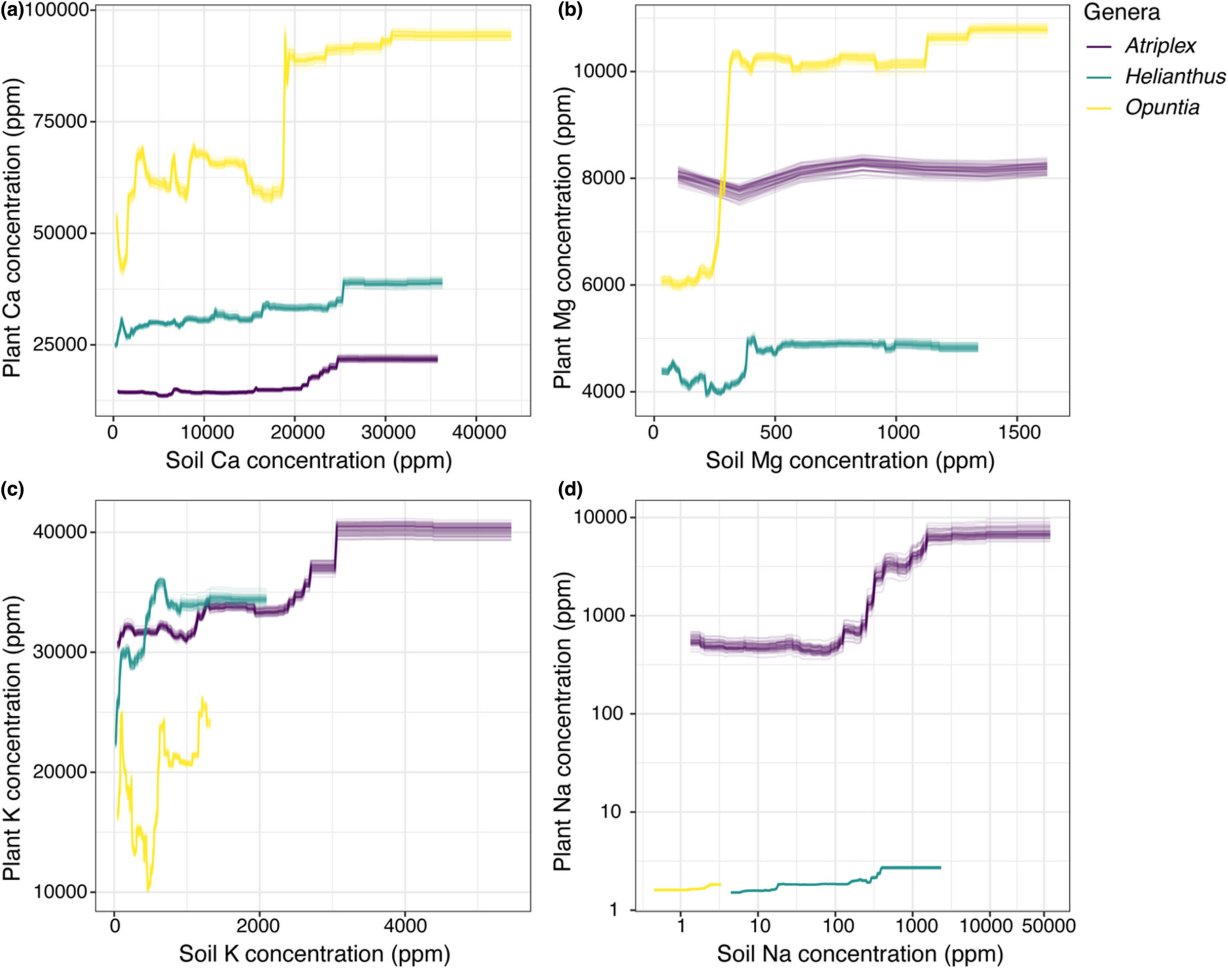
Responses of plant concentrations of (a) Ca, (b) Mg, (c) K and (d) Na for *Atriplex, Helianthus*, and *Opuntia* to soil cation concentrations (ppm). Each line represents a response curve estimated by a random forest model, which was fitted for each combination of plant genus and cation and repeated 50 times. Plant and soil Na are on a log_10_ scale.

Not all abiotic variables displayed the same influence on plant cation concentrations across the phytocationic landscapes (Figure 5). All models for *Atriplex, Helianthus*, and *Opuntia* shared MAT and soil cation concentration as the highest contributing variables influencing aboveground plant cation concentrations, albeit at different levels of importance (Figure 5, Table 4). Additionally, median R^2^ values for all models ranged from 0.57–0.94 (see Table 4), thus representing a high level of explained variation in plant cation concentrations. Mean annual temperature appeared to influence plant cation concentrations non‐linearly across taxa and cations, with *Opuntia* showing higher variation in responses as MAT increased. Sodium showed a unique response for *Helianthus*, where at around 18–19°C there is a sharp increase in plant Na concentrations, a pattern not observed in *Atriplex* and *Opuntia* (Figure 6). In terms of the influence of soil cation concentrations on plant cation concentrations, we found that in general plant cation concentrations increased non‐linearly, reaching a saturation point for all taxa and cations sampled, except in the case of K in *Opuntia* (Figure 7). We do not describe the response curves for other variables because they were not shared among all genera due to multicollinearity (Table 4).

**TABLE 4:** Model selection of environmental variable influences on plant cation concentrations obtained from the Random Forest analyses

Cation	Genus	Mean environmental variable relative importance												Median R^2^	Median Normalized RMSE
		MAT	MDR	TS	AP	PWM	PDM	PS	DNC	SPE	E	SpH	SC		
Ca	*Atriplex*	−0.009	0.36					0.07	0.14			0.26	0.44	0.93 ± 0.004	0.41 ± 0.007
	*Helianthus*	−0.01		0.004								0.27	0.24	0.92 ± 0.03	0.41 ± 0.004
	*Opuntia*	0.37											0.49	0.57 ± 0.006	0.74 ± 0.002
Mg	*Atriplex*	0.50	0.35					0.20	0.38			0.13	−0.16	0.91 ± 0.004	0.25 ± 0.002
	*Helianthus*	0.30		0.34								0.14	0.09	0.71 ± 0.004	0.49 ± 0.001
	*Opuntia*	0.63											0.56	0.69 ± 0.003	0.38 ± 0.001
K	*Atriplex*	0.18	0.32					−0.06	0.35			−0.21	−0.17	0.88 ± 0.003	0.39 ± 0.004
	*Helianthus*	0.45		0.19								0.13	0.47	0.72 ± 0.003	0.47 ± 0.001
	*Opuntia*	0.66											0.20	0.82 ± 0.002	0.37 ± 0.001
Na	*Atriplex*	0.28	0.43					0.42	0.48			0.18	0.54	0.92 ± 0.002	0.16 ± 0.001
	*Helianthus*	0.83		0.49								0.06	0.29	0.88 ± 0.002	0.22 ± 0.001
	*Opuntia*	0.41								0.33			0.29	0.94 ± 0.001	0.32 ± 0.003

*Note*: Environmental variables considered were MAT (mean annual temperature, °C), MDR (mean diurnal range), TS (temperature seasonality), MAP (mean annual precipitation, mm), PWM (precipitation on the wettest month, mm), PDM (precipitation on the driest month, mm), PS (precipitation seasonality), DNC (distance from the nearest effective marine source for cations, km), SPE (spatial predictors), E (elevation, m), SpH (soil pH, 1:1 water), and SC (soil cation concentration [ppm]). Soil cation concentration for Na was transformed using log_10_. The environmental variables' influences on overall plant cation variation are given in terms of their importance to the overall model output. For each genus, models are given horizontally and are constructed using the following example: *Plant Ca (ppm) ~ Mean annual temperature + Temperature seasonality + Soil pH + Soil Ca (ppm)*. For each best model selected the median R^2^, and median RMSE, and their respective standard deviations, for each model are given.

## 4 DISCUSSION

Plants across real‐world ecosystems in the southern United States, on average, follow the No‐Escape‐from‐Sodium hypothesis when exposed to variation in environmental Na across their ranges. This finding is congruent with patterns observed in laboratory studies (Santiago‐Rosario et al., 2021). In contrast, the phytocationic landscapes of Ca, Mg, and K do not closely follow soil concentrations of these elements. Even though Ca, Mg, and K concentrations in plant tissues are about as equally predictable as Na concentrations in plant tissues from a suite of environmental variables, Ca, Mg, and K appear to be considerably more homeostatically regulated than is Na in all three genera of plants considered in this study (Table 3).

### 4.1 Plant cation concentrations depend on the environment

Sodium is an unusual biotically important cation for plants as there is no apparent metabolic or structural function known for most plants (Benito et al., 2014; Kronzucker et al., 2013; Pardo & Quintero, 2002). Even so, plants have multiple mechanisms to manage tissue Na′s presence, example, ionic vacuole accumulation (Apse et al., 1999; Apse & Blumwald, 2007), salt gland extrusion (Dassanayake & Larkin, 2017), among other high energy‐consuming mechanisms (Kazachkova et al., 2018; Pantha & Dassanayake, 2020). These mechanisms help to prevent overall tissue toxicity, thus promoting survival. In any case, plants generally cannot escape having to cope with Na, especially in environments where its availability is expected and persistent (Santiago‐Rosario et al., 2021), such as near coasts and in arid environments.

Plant Na in *Atriplex* and *Helianthus* appeared to follow similar accumulation patterns, with plants having higher amounts of Na than adjacent soils (Figure 3d). However, *Opuntia* aboveground tissues appeared to share similar amounts of Na with the soil, possibly indicating an unusual strategy (Figure 3d). Moreover, the coefficient of variation in plant Na tissue concentration was approximately 40% for all genera, with *Helianthus* and *Opuntia* having higher variation in plant tissues than soil Na concentrations (Figure 3d). Variation in plant tissues of *Atriplex* was slightly lower than soil Na concentrations, which might reflect the halophytic nature of the family Amaranthaceae and the use of Na as a possible osmoticum in this genus (Glenn et al., 1994; White et al., 2017). Nevertheless, plant Na variation was substantially higher across all taxa than Ca, Mg, and K, suggesting less homeostasis for Na among plants in real‐world settings.

Our results also indicate weak to no homeostasis of Na by plants in real‐world ecosystems, which is consistent with the diversity of ways plants cope with substrate Na. In most plants, physiological mechanisms that mediate internal Na concentration rely on complex pathways and energetically expensive mechanisms impacting growth, metabolism, and performance as substrate Na increases to toxic levels (Apse & Blumwald, 2007; Cheeseman & Wickens, 1986a; Cheeseman & Wickens, 1986b; Maathuis, 2014; Munns & Tester, 2008; Pantha & Dassanayake, 2020). Although the genera sampled for this study are widespread across the southern United States, they only represent a small sample of plant phylogenetic diversity, suggesting that an expansion of taxa collected with the aim of understanding ecological stoichiometric patterns would be useful. Additionally, to better understand the mechanisms giving rise to patterns observed in the field, coupled laboratory and field experiments designed to manipulate substrate cations and gauge plant cation stoichiometric response across multiple plant taxa would be informative (Santiago‐Rosario et al., 2021).

Concerning the phytochemical landscapes of cations, their formation and maintenance are highly dependent on environmental factors, such as cation concentrations in the soil, among other environmental conditions (Figure 5). Soil cationic concentrations and soil pH levels interactively influence cation acquisition by plants; in most cases we found these variables to have high importance across models. A surprising component that influenced the models in a varied but important way was MAT, with *Opuntia* showing the highest degree of responses, suggesting that the genus might be particularly sensitive to temperature for plant Mg and K concentrations (Figure 6). MAT has been shown to have a dominant influence on overall moisture availability in soils across space and geological time despite precipitation amounts (Chevalier & Chase, 2016). Aridity, and drought conditions, have a substantial correlation with increasing cation concentrations in soils, especially for Na (Mukhopadhyay et al., 2021).

Considering plants' relationships with Na, the lack of homeostasis and the amount by which abiotic variables explained plant tissue concentration variation in our study reflect the influence of the environment on plant Na concentrations across the landscape. Both the lack of homeostasis and environmental influences on plant Na accumulation can be used to predict the phytochemical landscape of Na at different geographical scales. This is especially important in light of ongoing coastal salt intrusion and natural deposition (Dasgupta et al., 2015; National Atmospheric Deposition Program [NRSP‐3], 2020; Rahman et al., 2018), soil salinization due to poor irrigation practice, and drought (Ivushkin et al., 2019; Mukhopadhyay et al., 2021; Shahid et al., 2018), deicing road salt deposition across urban and rural areas (Bryson & Barker, 2002; Heintzman et al., 2015; Hintz et al., 2022; Mitchell et al., 2020; Snell‐Rood et al., 2014), among other anthropogenic influences on plants in the future (Konkel, 2016).

### 4.2 The phytochemical landscapes of Ca, Mg, and K appear to be at least partially controlled by homeostasis

Homeostasis plays a prominent role in some phytocationic landscapes, and responses differ substantially among genera (Wang et al., 2019). Calcium, Mg, and K were found at higher concentrations in plant tissues than in adjacent soils across all genera sampled. Plants apparently regulate these cations because of their essential biochemical and physiological functions (see Table 5). The variation of these cations in plant tissues differs substantially from Na; variation in Ca, Mg, and K is extremely low (coefficient of variation approximately 5% in all three genera; Figures 1 and 2). Although Ca, Mg, and K displayed higher homeostatic levels than Na, the concentrations varied among genera (Gilroy et al., 1993; Leigh, 2001; Tang & Luan, 2017). In *Helianthus*, Ca and Mg displayed homeostasis (according to Sterner and Elser's (2002) definition), and Mg and K appeared to be kept at high levels of homeostasis in *Atriplex* (Table 3). For the genera mentioned above, soil Ca, Mg, and K concentrations do not substantially influence plant concentrations of the same ions other than being the sole or primary source of the element (Farago, 1994).

**TABLE 5:** Review of cation function in plants

Cation	Function in plants
Calcium (Ca)	Cell wall stabilization ○ Structural component of the cell wall middle lamella Cell extension and secretory processes ○ Promotes cell elongation in roots, shoots, and pollen tubes ○ Aids in exocytosis of cell wall materials or secretion of mucilage. Membrane stabilization Maintaining homeostasis ○ Cation‐anion balance ○ Osmoregulation As a second messenger of environmental stress ○ Variation in Ca concentration in the cytosol regulates cell division, cell elongation, among other cellular responses, in response to environmental influences and/or plant phenology
Magnesium (Mg)	Plays a major role in photosynthesis ○ Central atom of the chlorophyll molecule Maintaining homeostasis ○ Promoted enzymatic regulation ○ Regulates cellular pH ○ Regulates cation‐anion balance Protein synthesis ○ Aggregation of ribosomal subunits
Potassium (K)	Osmoregulation ○ Maintains osmotic potential of cells and tissues ○ Maintains turgor pressure ○ Promoted stomatal movement by controlling guard cell osmotic pressure ○ Influences photonastic and seismonastic movements Homeostasis maintenance ○ Facilitates stabilization of cytosol pH aiding enzymatic activation and reaction ○ Cation‐anion balance ○ Promotes phloem transport of sucrose and other carbohydrates Stimulates protein synthesis by aiding protein translation processes ○ Promotes accumulation of soluble N compounds such as amino acids, amides, and nitrates Supports photosynthesis ○ Promotes stomatal regulation ○ Stimulates CO_2_ fixation ○ Control respiration rates

*Note*: Summarized from White & Broadley, 2003, Broadley et al., 2012, Hermans et al., 2013, Benito et al., 2014, Dechen et al., 2015, Nieves‐Cordones et al., 2016, Tang & Luan, 2017, Pandey & Mahiwal, 2020, and Sardans & Peñuelas, 2021.

Homeostasis might explain why, on average, the importance of the climatic variables considered in this study differed considerably in explaining the variation in plant tissue Ca, Mg, and K across the geographic range sampled (Figure 6, Tables 3 and 4). The role that Ca, Mg, and K play in plants varies greatly, from a fundamental structural component to metabolism and enzymatic reactions (Table 5). Therefore, the performance of plant tissues is tightly linked to the acquisition and maintenance of these elements, among others (Alemán et al., 2009; Sardans & Peñuelas, 2021; Tang & Luan, 2017; White & Broadley, 2003; Xu et al., 2020).

Because of anthropogenic global warming and increases in atmospheric CO_2_ concentrations, plant quality and stoichiometric mechanisms might be highly impacted, especially in highly regulated elements such as Ca and Mg. Evidence suggests warming conditions and increasing CO_2_ concentrations will affect the environmental availability of some essential elements and their regulation by plants (i.e., N and P) unevenly across their ranges (Dijkstra et al., 2012; Gu et al., 2017). Moreover, the nutrition dilution hypothesis posits that increases in atmospheric CO_2_, water availability, and temperature promote increased carbohydrate production in primary producers resulting in increases in plant biomass accumulation with low foliar nutrient quality, which in turn promotes a decline in herbivore abundance (Welti et al., 2020). Whether these stoichiometric patterns hold across other micronutrients and nonessential elements across plant taxa and different habitats remains unquantified.

Among plants' essential elements, such as Ca, Mg, and K, host‐specific herbivores should encounter a relatively consistent concentration of these elements across their ranges, yet no such patterns were observed for Na. Na is an essential element for animal metabolism and development (National Research Council [U.S.]., 2005) and its highly variable distribution across the phytocationic landscape might influence animal communities disproportionately. For instance, low levels of plant Na concentrations promote salt‐seeking behaviors in animals (i.e., *collpas* and salt lick visitation, increase in carnivory, among others) (Boggs & Dau, 2004; Bravo et al., 2010; Burger & Gochfeld, 2003; Clay et al., 2017; Holdø et al., 2002), whereas high levels of plant Na concentrations have generated unique herbivore adaptations to prevent salt‐induced stress encountered in some halophytic plant taxa that accumulate Na in plant tissues to evade herbivory (Kenagy, 1973; Renault et al., 2016). Yet, the mechanisms by which Na variation influences animal behavior across natural settings remain incompletely studied, especially when considering herbivorous species with large ranges.

### 4.3 Plants share similar Na concentrations the closer they are to each other

Sodium's high variability differs geographically, especially across soil Na gradients and proximity to coastlines with persistent salt deposits from marine aerosols (Borer et al., 2019; Bravo & Harms, 2017; Doughty et al., 2016). Not surprisingly, plant Na concentration exhibited strong spatial autocorrelation across all genera sampled, emphasizing the weak homeostatic regulation plants have for this cation along with the environmental influence on plant Na acquisition (Figure 3, Table 2). Individuals closer to each other share similar levels of tissue Na across a heterogeneous landscape. Moreover, Ca, Mg, and K, patterns of spatial autocorrelation were complex, albeit mostly weak across genera (Table 2). Potassium showed modest spatial autocorrelation only in *Atriplex* (Table 2). A similar pattern was observed across several plant families sampled geographically broadly in China, where K and Na showed strong spatial autocorrelation across leaf tissues, but Ca did not, thus suggesting that these general patterns might be shared globally across plant taxa (Zhang et al., 2012).

## 5 CONCLUSION

Our study illustrates the utility of focusing attention on the formation and maintenance of the phytochemical landscapes of essential (i.e., Ca, Mg, and K) and generally nonessential elements to plants, such as Na. The No‐Escape‐from‐Sodium hypothesis was supported across field‐collected plants, which suggests that plant tissue concentrations tend to reflect Na in the substrate, similar to patterns observed in controlled settings (Santiago‐Rosario et al., 2021). The differences in cation variation and spatial autocorrelation observed in this study appear to be linked to homeostatic regulation, or lack thereof, depending on elemental essentiality to plants. Thus, identifying general phytochemical patterns of essential and nonessential elements to plants across the landscape represents a key step toward better understanding biogeochemical cycles and their effects on trophic‐level interactions and ecosystem dynamics (Hunter, 2016; Sterner & Elser, 2002). Expanding this type of research to other essential and nonessential elements, other taxa, and additional geographic locations would broaden our understanding of the evolutionary and biogeographic processes that give rise to phytocationic landscapes.

## FUNDING INFORMATION

This study was supported by the Texas Ecological Laboratory Program.

## CONFLICTS OF INTEREST

The authors declare no conflict of interest.

## Data Availability

The data that support the findings of this study are openly available in Dryad at https://doi.org/10.5061/dryad.2bvq83bs2.
